# Characteristics of community-based exercise programs for community-dwelling older adults in rural/regional areas: a scoping review

**DOI:** 10.1007/s40520-022-02079-y

**Published:** 2022-02-12

**Authors:** Samantha Fien, Corey Linton, Jules S. Mitchell, Daniel P. Wadsworth, Helen Szabo, Christopher D. Askew, Mia A. Schaumberg

**Affiliations:** 1 School of Health, Medical and Applied Sciences, Central Queensland University, Mackay, Australia; 2 School of Health and Behavioural Sciences, University of the Sunshine Coast, Sippy Downs, Australia; 3 Thompson Institute, University of the Sunshine Coast, Birtinya, Australia; 4 School of Nursing, Midwifery and Paramedicine, University of the Sunshine Coast, Sippy Downs, Australia; 5 Sunshine Coast Health Institute, Sunshine Coast Hospital and Health Service, Birtinya, Australia; 6 Sunshine Coast Council, Birtinya, Australia; 7 School of Human Movement and Nutrition Sciences, The University of Queensland, Brisbane, Australia

**Keywords:** Older adult, Physical function, Psychosocial health, Regional/rural

## Abstract

Community-based exercise programs for older adults play a potentially important role in mitigating the decline in functional outcomes, body composition, psychosocial outcomes, and cardiovascular health outcomes that commonly occurs with advancing age. There is a limited understanding of the characteristics and effectiveness of community-based exercise programs, particularly when those programs are offered outside metropolitan areas. Rural/regional settings face unique challenges, such as limited access to equipment/resources, transportation, and services, as well as significant costs to run programs. The objective of this scoping review was to characterise studies in the field that have aimed to implement community-based programs in settings identified as rural / regional. A secondary aim was to establish guidance for future exercise programs in this setting and highlight future research directions. A total of 12 studies were conducted in settings identified as rural/regional areas in various countries across the world were included. Of the included studies, five were randomised controlled trials. The majority of included studies reported on functional outcomes (83%) and psychosocial outcomes (75%), yet only 42% reported body composition, 17% reported cardiovascular health and 17% reported dietary outcomes. Low male representation was observed, with women outnumbering men in 7 of 12 studies. There was also minimal investigation of qualitative outcomes in existing community-based exercise programs in rural/regional settings, presenting a key gap for future research to address. Study Protocol: https://osf.io/txpm3/. Date of registration: 20 July 2020.

**Supplementary Information:**

The online version contains supplementary material available at 10.1007/s40520-022-02079-y.

## Introduction

Ageing is associated with an increased risk of chronic disease, profoundly affecting the independence, mobility, and quality of life of older adults [1]. Globally, the most recent data estimate between 60 and 75% of adults over the age of 65 will be diagnosed with one or more chronic diseases [2–5]. Aside from the adverse effects on individual physical and cognitive functions, at the societal level, the financial burden of chronic disease management and treatment is profound, as evidenced by the United States expenditure constituting approximately 17% of GDP [6]. With the ageing population worldwide expected to double by 2050 [7], this issue will undoubtedly be compounded. This presents an unsustainable pressure on expenditure within the health system, that if to be mitigated, requires immediate attention. Interestingly, according to the models of Scott et al. [5], interventions targeted at reducing the impact of ageing on functional decline and dependence, have the potential to save US $38 trillion globally in 1 year [5].

Similar to many health-related issues, the challenges associated with an ageing population and chronic disease burden are likely to be exacerbated in rural/regional areas owing to the limited access to healthcare professionals and resources [8, 9]. What-is-more, exercise interventions can have profound results at an individual and societal level, for example in maintaining cognition, physical function, and wellbeing such that older adults may be able to lead a healthier, fulfilling, and productive life whilst remaining active contributors to their community [10–12]. Accordingly, there is a significant need to develop and implement more effective, and sustainable lifestyle interventions to manage, treat, and most desirably, prevent chronic diseases or their progression. Exercise presents as one of the most easily modifiable lifestyle factors with considerable evidence demonstrating its efficacy in mitigating the rate of age-related decline in physical, mental, and psychosocial health [13]. Whilst these positive benefits of exercise are well-documented [14], physical inactivity has remained high in the developed world [15], particularly in older adults [16].

Community-based group exercise programs are one approach that aim to encourage, enable, and engage older adults to participate in regular, appropriate, and health-promoting exercise. Importantly, these programs have the added potential to stimulate social engagement amongst members of the community; a factor known to improve health outcomes in older age, in and of itself [17–19]. This is achieved through maximising accessibility (sessions are held at community halls and local gyms); and financial affordability (sessions are free or low-cost). Previous systematic reviews of community-based exercise programs for older adults have shown that such programs can promote regular physical activity and improve general health outcomes [4, 20, 21]. In particular, improvements in mobility and physical function outcomes, and how they relate to reductions in frailty and falls risk, have consistently been reported as a common focus in the available research [4, 20].

A systematic review of community-based exercise programs in specifically rural/regional areas reported similar findings [22]. All seven studies included in that review incorporated low-to-moderate intensity exercise (e.g., tai-chi, balance, aquatics, yoga), and this led to positive changes in various physical and functional outcomes (e.g., balance, walking, strength, aerobic capacity) in five studies [23–27]. Notably, one study included a qualitative interview measure to assess perceived changes following the exercise program [27], with improvements in mental outlook reported. Despite the depth of existing literature, significant variability in study design, population characteristics, exercise program (i.e., type, duration, intensity, delivery), location, adherence levels, and outcome measures have been identified as key limitations [4, 22, 28]. Furthermore, because most research has focused on physical and functional outcomes, there is little known about the effect that rural/regional community-based exercise programs can have on perceived psychological and psychosocial outcomes in older adults.

Consequently, the current understanding of what factors influence the effectiveness, acceptability, and successful implementation of community-based exercise programs in older adults living in rural/regional areas is limited. The objective of this scoping review is to identify and summarise the characteristics and outcomes of existing community-based exercise programs for community-dwelling older adults living in rural/regional areas. In doing so, this will provide guidance to existing and future programs, and highlight future research directions [29].

This scoping review addresses the following questions:

1. What are the aims and characteristics (exercise modality, delivery mode, duration, and supervision) of community-based exercise programs in settings that are identified as rural/regional areas?
2. What are the demographics and health status of older adults participating in community-based exercise programs in settings that are identified as rural/regional areas?
3. What types of outcomes (primary or secondary) have been reported?
4. What factors/variables are associated with successful rural/regional community-based exercise programs?

Addressing these aims will support the development and implementation of resources and evidence-based approaches to support the successful implementation and evaluation of community-based programs for older adults residing in rural/regional areas.

## Methods

### Protocol and registration

Scoping review methodology was chosen as it is the most appropriate method to identify key characteristics related to the topic of investigation: community-based exercise programs for community-dwelling older adults living in rural/regional areas [30]. This review followed the Joanna Briggs Institute (JBI) Scoping Review Methodology [31] and is reported according to the guidelines outlined in the PRISMA Extension for Scoping Reviews (PRISMA-ScR) [32]. Prior to commencing this review, the research team developed and published a scoping review protocol available at (https://osf.io/txpm3/). The protocol described the planned approach and full methods for this scoping review, and so these are described briefly in this paper.

### Eligibility criteria

Included studies reported an evaluation / assessment of community-based exercise programs in settings identified as rural/regional areas with older adults, aged 65 years or older, which measured one, or a combination of, physical, psychological, or qualitative outcomes. There were no restrictions for the exercise programs in terms of delivery with the inclusion criteria being programs that targeted independently living older adults in settings identified as rural/regional areas. Given our focus on healthy older adults, studies that specifically targeted the treatment or rehabilitation of a diagnosed disease, condition, or injury (e.g., dementia, stroke, cancer, diabetes mellitus, and osteoarthritis) were excluded. No publication date restriction was applied, however, citations generated were limited to English studies. The study designs included were experimental (randomised controlled trials (RCTs), quasi-RCTs, non-RCTs), quasi-experimental (controlled before after, interrupted time series), and observational (cohort, case control, cross-sectional). Qualitative study designs (for example case study designs) as well as reviews, grey literature (for example dissertations and government reports), text, and opinion papers were included.

### Information sources and literature search

A comprehensive literature search was conducted, which was not time-limited, in Cochrane Library (Wiley), PubMed (NLM), Scopus (Elsevier), and Web of Science (Clarivate). The search terms included community-based, independent, older adults, over 65, exercise, physical activity, rural, and regional. For studies that did not explicitly stipulate if the location was rural/regional, they were excluded from this review. To ensure a more comprehensive search, additional terms were incorporated to target rural/regional locations (e.g., semi-urban, non-metro, county). All search strategies, both initial and revised, for the other databases are available from the corresponding author upon request. A secondary search of the reference lists of the 12 included studies was carried out by two members of the team (JM and SF) to identify any additional studies.

### Study selection process

A preliminary screen of 25 selected studies was conducted to establish a level of agreement in accordance with the standard set by JBI [31], and to flag any issues with the inclusion/exclusion criteria. As the level of agreement was greater than 90%, the reviewers completed screening for the remaining studies according to the eligibility criteria outlined in the protocol. Studies were screened for inclusion through a two-step process; the first step screening title/abstract and the second step assessing full-text articles, with any disputes between the two reviewers (JM and SF) resolved through discussion, or failing this, using an independent third reviewer (DC).

The following information was extracted: author, year and country of publishing, study design, study aim, follow-up timepoints, inclusion of a rural/regional definition, location of program, inclusion of a focus group, sample size, female percentage, reporting of chronic diseases in the sample, intervention duration, frequency of sessions, duration of session, exercise type, specific exercises, delivery mode, supervision and if that included a health professional, comparison/control group, adherence and influence of adherence reported. The main outcomes identified were extracted in tabular format: functional outcomes, body composition, psychosocial outcomes, cardiovascular health, and dietary outcomes.

## Results

### Literature search

**Fig. 1:**
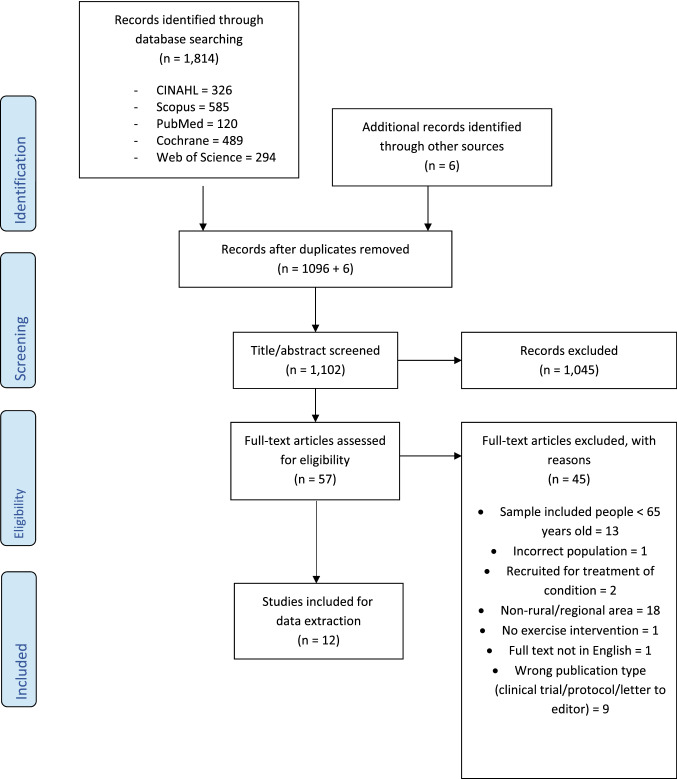
PRISMA flow diagram describing record inclusion through different stages of screening; includes number of records identified, included and excluded, and the reasons for exclusion

From the database and grey literature search, 1814 citations were generated. Following duplicate removal, title/abstract and full-text screening against the eligibility criteria, 12 articles were included in the final review. A breakdown of the search results, duplicate removal, and reasons for study exclusion are reported in Fig. 1.

### Terminology and definition of rural/regional

Due to there being no conclusive definition for rural/regional locations for this review, rural/regional was defined as follows: (1) the article needed to specifically include the terminology “rural/regional/other related term location (i.e., specify that the study was not conducted in a major city centre)”. The characteristics of each study location can be found in Table 1. The used term to describe the location was rural (100%). No studies provided a definition of what constituted a regional area. Most studies (58%) reported where the delivery of the exercise intervention took: three in a community hall / centre; one at social club; one in the local gym; one in the village; one at home. However, five studies did not report the details.

**Table 1:** Characteristics of included studies

Authors & year of publication	Location	Term used—description of location	Study design	Study aim	Follow-up timepoints	Definition of rural	Location of program	Focus group
Jang et al. 2018 [33]	Pyeongchang, South Korea	Rural	Quasi-experimental: Pre-post intervention (non-randomised)—Independent groups	Promote self-exercise using a wearable device	6 months and 13 months	N/A	N/A	N/A
Jindo et al. 2016 [34]	Ibaraki prefecture, Japan	Rural	Quasi-experimental: Pre-post intervention (non-randomised)—Independent groups	Determine if wearing a pedometer influences the effectiveness of square-stepping exercise program	11 weeks	N/A	Community Centre	N/A
Jindo et al. 2017 [35]	Kasami City (Ibaraki Prefecture), Japan	Rural	Quasi-experimental: Pre-post intervention (non-randomised)	Determine how daily life physical activity effects square stepping exercise program	11 weeks	N/A	Community Centre	N/A
Lin et al. 2006 [36]	Shin-Sher Township (Taichung County), Taiwan	Rural	Quasi-experimental: Pre-post intervention (non-randomised)	Determine the effect of a tai-chi program on falls, balance, gait and fear of falling	52 weeks	N/A	In Village	N/A
McMahon et al. 2016 [37]	Itasca County, Minnesota	Rural	Randomised controlled trial; repeated measures	To assess the feasibility of a motivational intervention and falls reducing physical activities	8 weeks	N/A	N/A	N/A
Muscari et al. 2010 [38]	Pianoro, Northern Italy	Rural	Randomised controlled trial	To evaluate the effects of endurance exercise training (EET) on the cognitive status of healthy community-dwelling older adults	Weekly, 12 weeks, 24 weeks, 36 weeks and 52 weeks	N/A	Local gym	N/A
Okumiya et al. 1996 [39]	Kahoku, Japan	Rural	Randomised controlled trial	Evaluate the effects of exercise on neurobehavioral function	26 weeks	N/A	N/A	N/A
Snapp et al. [40]	Southern Illinois	Rural	Quasi-experimental: Pre-post intervention (non-randomised)	To test the overall balance of older adults who participated in a modified 10-step Tai Chi Chuan program	8 weeks	N/A	N/A	N/A
Sowle et al. 2017 [41]	Iowa, USA and surrounding communities	Rural	Quasi-experimental: Pre-post intervention (non-randomised)	To observe the effect of a community-based exergaming program on physical activity readiness-to-change and self-efficacy among rural-residing older adults	8 weeks	N/A	Churches, retirement and assisted living communities, community centres, wellness centres, and offices of Iowa State University	N/A
Tarazona-Santabalbina et al. 2016 [42]	La Ribera, Spain	Rural	Randomised controlled trial	To determine if a supervised-facility multi-component exercise program can reverse frailty and improve functionality; cognitive, emotional, and social networking; as well as biological biomarkers of frailty	2 -weeks	N/A	Social club	N/A
Wang et al. 2014 [43]	Chiayi Country, Taiwan	Rural village	A prospective quasi-experimental design	Observe the effect of a CBHP on change of lifestyle, physiological indicators and depression score among seniors	24 weeks	N/A	N/A	N/A
Yates and Dunnagan 2001 [27]	Southwest Montana	Rural	Randomised controlled trial	Determine the effectiveness of a home-based falls risk reduction program	10 weeks	N/A	Home	N/A

### Study characteristics

Studies were published between 1996 and 2018, and were conducted in both western and non-western cultures. A large proportion of studies were conducted in the United States of America (*n* = 4, 33%), Japan (*n* = 3, 25%), and Taiwan, *(*n = 2, 16%), with the remaining locations consisting of Italy (*n* = 1, 8.3%), Spain (*n* = 1, 8.3%), and South Korea (*n* = 1, 8.3%). Four studies were randomised controlled trials [37–39, 42] and seven were quasi-experimental studies [33–36, 40, 41, 43]. There were no observational or qualitative studies included. A further summary of the study characteristics can be found in Table 1.

### Population characteristics

The characteristics of the sample population for each study can be found in Table 2. Across all studies, the age of participants ranged from 65 to 90 years, with a mean of 74.73 (SD = 5.35). The sample sizes ranged from 22 to 520 participants. Females constituted a larger portion of the sample size in ten studies: with female sample percentage below 50% in two studies [34, 38]. All included participants (including the comparison group) were relatively healthy (i.e., not requiring a carer, currently hospitalised, or specifically recruited for rehabilitation or treatment) and living independently within the local community.

**Table 2:** The characteristics of the sample population for included studies

Authors & year of publication	Sample characteristics					
	Sample size	Age range (mean)		% Female (*n* =)		Chronic disease
		Intervention	Comparison	Intervention	Comparison	
Jang et al. 2018 [33]	22 (Control = 11)	66–70 (68.6)	68–76 (72.5)	17% (3)	45% (5)	None reported—although included prefrail group screened using the cardiovascular health frailty phenotype criteria
Jindo et al. 2016 [34]	68 (Control = 34)	67–73 (70.0)	66–74 (70.0)	91.2% (31)	91.2% (31)	None reported—ambulant and living independently
Jindo et al. 2017 [35]	46	66.6–73.6 (70.1)	N/A	87% (40)	N/A	None reported—ambulant and living independently
Lin et al. 2006 [36]	88	65 + (upper range and mean not specified)	N/A	68.2% (60)	N/A	Yes, 68.2% reported at least 1 comorbid condition (not specified)
McMahon et al. 2016 [37]	30 (Control = 14)	78.9–88.3 (83.6)	78.9–88.3 (83.6)	93.3% (15)	93.3% (13)	None reported
Muscari et al. 2010 [38]	120 (Control = 60)	66.3–71.3 (68.8)	66.8–72.4 (69.6)	47.7% (28)	50% (30)	5% COPD, 73.3% Hypertension, 60.8% Hypercholesterolaemia
Okumiya et al. 1996 [39]	42 (Control = 21)	75–87 (78.8)	75–87 (78.8)	57.1% (12)	57.1% (12)	None reported
Snapp et al. 2009 [40]	19 (Control = 9)	> 65 (85)	> 65 (85)	80% (8)	100% (9)	None reported—Participants living at an assisted living facility or an independent living facility
Sowle et al. 2017 [41]	265	65–85 +	N/A	83.4% (221)	N/A	None reported—ambulant and living independently
Tarazona-Santabalbina et al. 2016 [42]	100 (Control = 49)	76.1–83.3 (79.7)	76.6–84 (80.3)	56.9% (29)	51% (25)	77% Hypertension, 46% Hyperlipidaemia, 8% COPD
Wang et al. 2014 [43]	520 (Group 1* = 431)	68.53–80.81 (74.67)	71.45–82.01 (76.73)	65% (280)	68.5% (61)	None reported
Yates and Dunnagan 2001 [27]	37 (Control = 19)	67–90 (76)	69–88 (78)	72.2% (13)	68.4% (13)	None reported

*Group 1 = nursing home group intervention vs Group 2 = community-dwelling intervention

### Exercise intervention characteristics

The characteristics for each exercise intervention are presented in Tables 3 and 4.

**Table 3:** Methodology for intervention groups of the included studies

Authors & year of publication	Exercise group								
	Intervention duration	Total sessions (frequency)	Session duration (minutes)	Exercise type	Delivery mode	Supervision	Supervised by health professional	Adherence—range (mean)	Influence of adherence
Jang et al. 2018 [33]	13 month (6 month coaching, 1-month rest, 6-month self-management)	No sessions—coaching consisted of notification messages sent to wearable device with step count goals	N/A	Walking	Externally, via wearable device	Un-supervised	N/A	N/A	N/A
Jindo et al. 2016 [34]	11 weeks	11 (1/week)	90	Square-stepping	In-person group	Supervised	No	72.7%—100% (93.6%)	N/A
Jindo et al. 2017 [35]	11 weeks	11 (1/week)	90	Square-stepping	In-person group	Supervised	No	63.6%—100% (91.8%)	N/A
Lin et al. 2006 [36]	52 weeks	No specific session number was set (sessions offered 6 days/week)	60	Chen-style tai chi	In-person group	Supervised	No	49%—87% (63%)	Physical inability to get to the places where the group exercise was conducted
McMahon et al. 2016 [37]	8 weeks	8 (1/week)	90	Strengthening, balance retraining, flexibility, walking	In-person small group and encouraged externally	Supervised and Un-supervised	Yes	Not reported	N/A
Muscari et al. 2010 [38]	52 weeks	156 (3/week)	60	Endurance exercise training	In-person groups of ~ 20	Supervised	Yes	Not reported	N/A
Okumiya et al. 1996 [39]	24 week	48 (2/week)	60	Mix exercise group	In-person group	Supervised	Yes	59%-100% (86%)	Opportunity for social interaction
Snapp et al. 2009 [40]	8 weeks	24 (3/week)	30	10-Step Tai Chi	In-person group	Supervised	No	44%	N/A
Sowle et al. 2017 [41]	8 week	16 (2/week)	30–60	Life Program	In-person group	Supervised	No	58%	N/A
Tarazona-Santabalbina et al. 2016 [42]	24 week	120 (5/week)	65	Multicomponent Exercise Program and nutritional education	In-person group	Supervised	No	38.7%-55.7% (47.3%)	N/A
Wang et al. 2014 [43]	24 week	48 (2/week)	120	Seniors health promotion program delivered by senior nurses	In-person group	Supervised	Yes	Not reported	N/A
Yates and Dunnagan 2001 [27]	10 weeks	12 +	15	Fall risk education, home-based exercise programming (improving strength, coordination, balance, mobility through chair exercises), nutrition counselling, and environmental hazards education	In-person orientation, exercises encouraged at home	Un-supervised	N/A	72.20%	N/A

**Table 4:** The specific intervention method for included studies

Study	Exercise intervention				
	Type (e.g., aerobic or resistance)	Specific exercises	Duration (minutes)	Duration (minutes/ week)	Meets WHO guidelines
Jang et al. 2018 [33]	Walking	N/A	N/A	N/A	N/A
Jindo et al. 2016 [34]	Square-stepping	Warm-up (15 min), square stepping exercise (40 min), a recreational activity (20 min), and cool-down (15 min)	90	90	X
Jindo et al. 2017 [35]	Square-stepping	Warm-up (15 min), square stepping exercise (40 min), a recreational activity (20 min), and cool-down (15 min)	90	90	X
Lin et al. 2006 [36]	Chen-style tai chi	Warm-up (10 min), chen-style tai chi—13 movements; not specified (45 min), and cool-down (5 min)	60	N/A	N/A
McMahon et al. 2016 [37]	Strengthening, balance retraining, flexibility, walking	Knee extensor, knee flexor, hip abductor, ankle flexors, ankle dorsiflexors, knee bends, backwards walking, walking and turning around, sideways walking, tandem stance, tandem walk, one leg stand, heel walking, toe walk, heel-toe backward walk, sit to stair walking, head flexibility, neck flexibility, back extension, trunk flexibility, ankle movement	90	90	X
Muscari et al. 2010 [38]	Endurance exercise training	Cycle ergometer, treadmill and free-body activity, in variable order according to machine availability	60	180	
Okumiya et al. 1996 [39]	Mix exercise group	Warm-up (5 min), light aerobic exercise; walking, balance, game playing, stretching, range of motion exercises, muscle strengthening exercises (50 min) and cool down (5 min)	60	120	X
Snapp et al. 2009 [40]	10-step Tai Chi	Tai chi preparation- Raise arms, inhale. Sink elbows, hands follow, exhale. Part the wild horse main, white crane spreads its wing, brush knee and twist step, hands strum the lute, step back and whirl arms, grasp the birds tail (left and right), single whip, wave hands as clouds, single whip	30	90	X
Sowle et al. 2017 [41]	Life PROGRAM	Exercise using Xbox Kinect exergaming technology using Kinect Sports	30–60	60–120	X
Tarazona-Santabalbina et al. 2016 [42]	Multicomponent Exercise Program and nutritional education	Proprioception and balance exercises (10–15 min), aerobic training (initially at 40% of maximum heart rate increasing progressively to 65%), strength training (initially at 25% of 1 repetition maximum to 75%), and stretching	65	325	
Wang et al. 2014 [43]	Seniors health promotion program delivered by senior nurses	Education; how to: choose a healthy diet, maintain oral hygiene, prevent falls, engage in physical activity, self- protect, be responsible for health, manage stress, and use resources (90 min), mild exercise (30 min)	120	240	
Yates and Dunnagan 2001 [27]	Multicomponent Exercise Program and nutritional education	Fall risk education, home-based exercise programming (improving strength, coordination, balance, mobility through chair exercises), nutrition counselling, and environmental hazards education	15	N/A	N/A

**Exercise type:** Significant variations in type, delivery and duration of exercise were noted across the included studies. A total of six studies offered only a single exercise type [33–36, 38, 40], with six studies offering two or more exercise types. Of the studies with a single exercise type, square stepping (two studies), tai-chi (two studies) and aerobic training (two studies) were commonly reported followed by walking (one study), endurance exercise (one study). Of the multi-component exercise programs, two studies included combined balance/strength exercise, two studies combined aerobic/balance (with one being exergame), one fall-risk nutrition and hazard education, combined with strength, coordination, balance, mobility, and one study combined strength, balance, and flexibility exercises.

**Exercise delivery:** In most studies (10/12), exercise was the sole intervention. Three studies incorporated exercise as part of a multi-component intervention, alongside nutrition, counselling, and health seminars [27, 41–43]. Nearly all (11/12) exercise interventions were delivered in-person; of these, all 11 studies offered group exercise classes, and no studies offered individual exercise classes. Online, one-on-one exercise interventions were offered in two studies. The exercise was supervised in 83.3% of studies, with only 33.3% of the supervisors were university-trained exercise professionals (e.g., exercise physiologists, sports scientists).

**Exercise duration**: Program duration ranged from 8 to 56 weeks, with the vast majority (61.1%) between 8 to 12 weeks in duration. Exercise sessions were offered between 1 and 3 times per week, ranging from 15 to 120-min per session. Fifteen studies included pre- and post-program assessments, and three reported at least one follow-up assessment. Follow-up time-points ranged from weekly to 56 weeks.

**Adherence:** Program adherence was recorded in 13 studies. Of these, adherence greater than 80% was reported in 46% of studies (6/13), whilst three studies reported adherence below 60%. Three studies identified factors that influenced adherence, including injury (50%), physical inability to get to sites (25%) and time constraints (25%). Self-efficacy to participate in physical activity [41], and reduced cost [33, 43], were reported to influence adherence. In addition, the capacity to have individualised programs and education on self-management techniques [33]; a mix of age groups [37]. Notably, in one study that utilised a wearable device to promote physical activity, simplicity, ease of use, battery life, and affordability predicted high adherence [33].

**Qualitative data:** No qualitative data were reported for any of the included studies.

**Control groups:** Whilst the majority of the control group interventions had no details regarding the delivery mode of the intervention (*n* = 10, 55.56%), the remainder were a combination of in-person group sessions (*n* = 5, 27.8%), individual (*n* = 1, 5.56%), at-home (*n* = 1, 5.56%), and external—via wearable device (*n* = 1, 5.56%).

### Outcome measures

All outcome measures are summarised in Table 5. A total of 15 studies included physical function measures, with psychosocial measures reported in only 9 studies. Physical function measures included: single leg balance (4/12 studies), 30-s sit-to-stand (2/12 studies), and timed up-and-go (7/12 studies). In addition, physical activity was measured with step count (6/12 studies). Fear of falling was the main psychosocial outcome measured, being reported in two studies. Cognition was only assessed in three studies (via the Mini-Mental State Examination (MMSE)). No studies employed focus groups as a means of obtaining qualitative measures. Total fat mass was the main measurement of body composition (3/12 studies) and only two studies reported outcomes for cardiovascular health. Only two studies reported dietary outcomes, looking at a healthy diet and nutrition.

**Table 5:** Outcome measures for each included study

Authors & year of publication	Functional outcomes	Body composition	Psychosocial outcomes	Cardiovascular health	Dietary outcomes
Jang et al. 2018* [33]	Step count, gait, number of falls	Total fat mass	IPAQ, quality of life	N/A	N/A
Jindo et al. 2016* [34]	Step count, 30-s chair sit to stand, single leg balance, timed up and go, 5-m habitual walk, lower extremity physical function score, choice stepping reaction time	N/A	N/A	N/A	N/A
Jindo et al. 2017* [35]	Step count, 30-s chair sit to stand, single leg balance, timed up and go, 5-m habitual walk, choice stepping reaction time	N/A	N/A	N/A	N/A
Lin et al. 2006* [36]	Gait, number of falls, single leg balance	N/A	Fear of falling, IPAQ	N/A	N/A
McMahon et al. 2016* [37]	Short test battery	N/A	Acceptability questionnaire and indices of procedural consistency, community health activity model program for senior's questionnaire, perceived environmental support scale, social support for exercise questionnaire, goal attainment scale, index of readiness, index of self-regulation	N/A	N/A
Muscari et al. 2010* [38]	Power at cycle ergometer	BMI, total fat mass, waist circumference	MMSE, cognitive status	Heart rate at exercising, VO2 max	N/A
Okumiya et al. 1996* [39]	Timed up and go, functional reach	BMI	MMSE, cognitive status	N/A	N/A
Snapp et al. 2009 [40]	Balance scale	N/A	N/A	N/A	N/A
Sowle et al. 2017* [41]	N/A	N/A	Self-reported readiness to change, self-efficacy to overcome perceived barriers to physical activity, physical activity self-efficacy	N/A	N/A
Tarazona-Santabalbina et al. 2016* [42]	Grip strength, short test battery, timed up and go, 6-min walk test, number of falls, Barthel index, Lawton and Brody, Tinetti	BMI, total fat mass, abdominal, brachial and leg girths, lean mass	Quality of life, social support for exercise questionnaire, MMSE	N/A	N/A
Wang et al. 2014* [43]	N/A	Waist circumference	Community participation, health responsibility, geriatric health-promoting scale	Fasting blood sugar, total cholesterol, blood pressure	Healthy diet
Yates and Dunnagan 2001* [27]	Single leg balance, timed up and go, lower extremity physical function score, bicep endurance, scratch test, dorsiflexion left/right	N/A	Fear of falling, depression	N/A	Control for nutrition, nutritious food behaviour

^*^Indicates a significant change in a reported outcome

## Discussion

The overall aim of this scoping review was to investigate the characteristics and outcomes of community-based exercise programs for older adults living in rural/regional areas. Only 12 publications met the predetermined eligibility criteria, indicating the lack of research in the rural/regional context as well as the lack of qualitative/psychosocial outcomes. Approximately one-third of older adults live outside of major cities in Australia, Canada, and United States [44–46], whereby access to health services and community exercise programs is often limited due to access, travel, and affordability. Highlighting the importance of specific consideration for the needs of the rural/regional older adult population.

Achieving healthy ageing requires adequate healthcare systems; an integral part of which includes having long-term exercise and physical activity programs in place to prevent a litany of chronic and other diseases, and thus be proactive in improving the health and wellbeing of an increasing ageing population throughout the world [9] Indeed, Izquirdo et al. (2021) suggest that the current body of evidence makes it ‘unethical’ not to prescribe physical activity to older adults. There is an opportunity to address the inequity of health in rural/regional older adults by offering accessible forms of exercise. This scoping review has provided an opportunity to assess the characteristics of community-based exercise programs that are likely to be feasible and effective in rural/regional settings.

All interventions in the current review included low-to moderate intensity exercise; predominantly walking, square-stepping and Tai Chi. This finding is consistent with the systematic review by Moore et al. [22]. However, Moore and colleagues included studies from urban and rural communities (two out of the seven studies) [22], whereas the current review was specifically focussed on rural/regional communities. Vast variety in the type, delivery and duration of exercise interventions was found across the included studies. Most programs (72%) offered only a single exercise type, and the studies largely evaluated physical function (83%) rather than psychological measures (50%). Interestingly, a review of 19 systematic reviews relating to exercise interventions for older adults living in the community revealed that any modality of exercise whether multi-modal or single, were more effective at increasing physical activity levels than no intervention at all [47]. Regardless of the setting, community-based exercise programs should include a variety of multi-component exercises of a moderate intensity, both to align the program with WHO 2020 guidelines on physical activity and sedentary behaviour [48] and to provide the participants with variety and autonomy/choice. Of particular interest, in line with WHO 2020 guidelines on physical activity [48], 150–300 min of moderate intensity or 75–150 min of vigorous intensity was prescribed in four studies [38, 42, 43, 49] as referred to in Table 4. Alongside the WHO exercise guidelines, a strong recommendation for older adults to take part in multi-component exercise that includes functional balance and strength training on three or more days a week to reduce the risk of falls is also included [48]. Only two of those previous four studies met this additional requirement [38, 42].

A large proportion of these studies included in this review pre-date the use of video conferencing platforms. However, with the spread of COVID-19, the use of video teleconferencing for the delivery of exercise interventions to rural/regional areas have not yet been capitalised for the use of telehealth and online fitness programs [50]. Whilst telehealth does increase falls risk as supervision cannot assist participants if they are to lose their balance, there is still the need for older adults to participate in exercise to improve quality of life, functional independence, and health [51]. Telehealth exercise interventions may also be considered a positive for rural/regional areas, where access to professional services / supervision is thus improved, the need for travel is reduced, and less strain is placed on the healthcare system [51].

Consistent with two previous reviews, a key finding from this review was the limited number of studies that utilised valid and reliable measurement tools to measure the outcomes (e.g., functional outcomes) to quantify the benefits of community-based exercise interventions. In terms of functional outcomes, the use of valid and objective measures of functional ability (such as 30 s chair sit to stand, timed up and go, and a balance test) in combination with at least one validated body composition measurement, a psychosocial outcome, and a cognitive measurement would help to provide consistency with reporting of important outcomes. The main outcomes measured and reported were functional outcomes, which were reported in 10 of 12 studies. Whilst psychosocial, body composition, and cardiovascular health outcomes were also reported, the lack of qualitative investigation of psychosocial parameters known to influence health outcomes, such as loneliness, and the lack of assessment of other health-related behaviours such as dietary patterns, limits the conclusions that can be drawn about the effectiveness of exercise interventions for improving health outcomes of older adults in rural/regional areas.

Healthy ageing is multi-faceted and requires a holistic focus on all aspects of health and wellbeing, including functional, body composition, psychosocial, cardiovascular, and dietary outcomes. However, none of the published programs evaluated all the relevant measurable outcomes. Noting that none of the included studies evaluated a measurable outcome of social participation or loneliness. Loneliness reduces quality of life and is associated with a 29% increased risk of mortality and higher chronic disease occurrence [16]. Exercise can facilitate social participation; however, it may be limited in its reach among older adults living in rural/regional areas, potentially increasing health inequalities and vulnerability of this sector of the population [52]. In turn, by not placing exercise as an important factor in the model of healthy ageing, significant limitations are being imposed on older adults’ independence and quality of life [53, 54].

Overall, there is still a limited understanding of the characteristics and effectiveness of community-based exercise programs, particularly within rural/regional areas. These areas face unique challenges, such as limited access to equipment/resources, transportation, and services, as well as significant costs to run the programs, which are important considerations in the success of programs. In addition, outcome measures should be valid, reliable, and applicable to regional older adult populations and factors associated with wellbeing, longevity, and quality of life. This would enable a greater understanding of the clinical importance and real-life impact of an intervention or exercise program in areas of need.

**Fig. 2:**
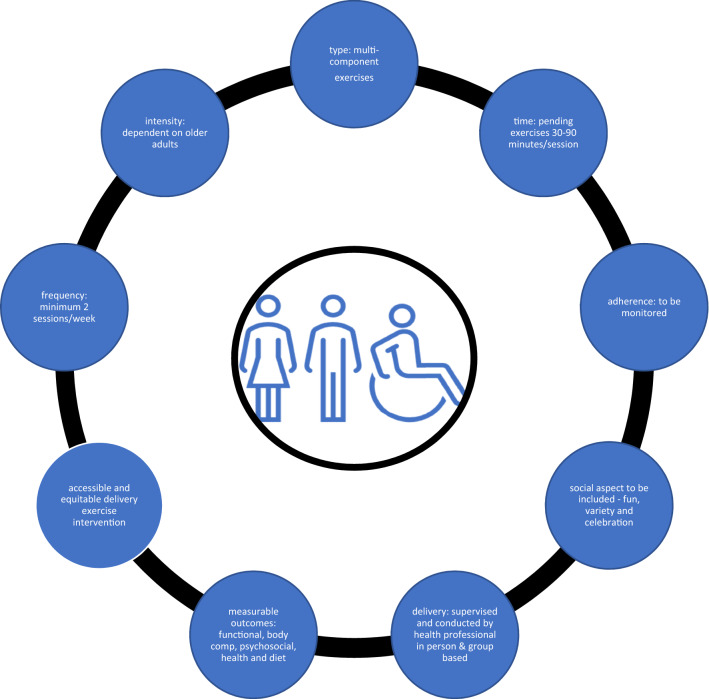
Variables to include in a successful rural/regional community-based exercise programs

Figure 2 presents recommendations from the included studies integrated with the authors’ suggestions for successful community-based exercise program delivery for older adults residing in rural/regional areas.

## Recommendations for future research

Future research is essential to investigate the effectiveness, acceptability, and successful implementation of community-based exercise interventions located in rural/regional areas. Of note, a significant finding of this review was that no studies clearly defined a rural/regional location. The assumption that a program is based in a rural/regional area based on minimal reported details, limits the specificity of the recommendations to programs running in rural/regional areas. Future research should include the location details of programs, particularly given the dynamics of program success that may be impacted with geographical location. We recommend that a definition of a rural/regional area is utilised.

In addition, future research must place strong consideration on participant recruitment to ensure representative samples, as most studies were limited by small sample sizes, high percentage of female participants, selection bias, exclusion of frailer older adults or those with dementia, and no blinding. More representative sampling will inform the development of relevant recommendations aimed at improving community-based exercise programs for older adults that consider the diversity of populations in regional/rural areas.

Finally, psychosocial outcomes were sparsely reported in study outcomes. Exercise can have profound psychosocial impacts on older adults [12, 55], whilst consideration of such behavioural elements can enhance program adherence, increasing participant motivation, self-efficacy and feelings of control [16, 56]. Such psychosocial improvements can in-turn lead to increasing general health, physical activity, social interaction and independence, concurrently enhancing mental wellbeing and Quality of Life [57]. Considering the pivotal impact that psychosocial factors such as wellbeing and depression have on the physical function and quality of life of older adults [58], the current authors strongly recommend that future research addresses this gap in the current literature.

## Limitations and gaps

This review may not contain an exhaustive list of community-based exercise interventions that may benefit the physical and psychosocial wellbeing of older adults living in rural/regional area. This study only investigated rural/regional community-based exercise interventions targeting older adults aged 65 years or older. As stated by Moore et al. [22] there is currently no international definition of ‘older adults’ and as stated by the UN there is currently no international definition for rural/regional areas [59]. Therefore, limiting our search to adults aged over 65 years and studies that explicitly stated by the authors in rural/regional areas studies including populations that may have been considered older in certain locations may have been overlooked [22]. Studies should specifically identify ‘rural/regional settings’ as a focus, and to make this clearer as to the location of their study. There is a real need for more focused research that contrasts the needs of regional versus non-regional communities where one approach may not be suitable for all participants.

Several studies did not specify what target population was being included in the study or included those below the age of 65 years; and/or did not include a comparison group as a control group or of a lesser intensity than the exercise group. This highlights the need for researchers to clearly report characteristics of intervention participants and to include a control/comparison group when undertaking evaluations of community-based exercise interventions or programs in rural/regional areas.

Most studies in this review reported at least one limitation to their findings (88%); only two studies did not report on any limitations (12%). Most commonly, small sample size was noted as a limitation, reported in seven studies (39%). Other common limitations included lack of a control group (28%), not accounting for potential confounding variables such as social/psychological factors (28%) and poor study design (28%). These limitations present exciting and important avenues for future investigation in this area.

## Conclusion

Evaluation of the effectiveness of rural/regional community-based exercise programs was primarily focused on physical and functional health outcomes, with almost 90% of included studies reporting such measures. Low male representation was common, with women outnumbering men in most studies, presenting the need for recruitment strategies that target men as well as women. Finally, there has been minimal investigation of qualitative outcomes in existing community-based exercise programs, presenting a key gap for future research to address. The importance of effective community-based exercise programs is vital for the physical and psychosocial health of older adults living in rural/regional locations in an ageing international climate.

## Supplementary Information

Below is the link to the electronic supplementary material.

Supplementary file1 (PDF 118 KB)

Supplementary file2 (PDF 99 KB)

## Data Availability

All data used for this review are presented in tables, figures, or supplementary files. Further queries can be submitted to the corresponding author.
