# Supplementary material for: Characteristics of community-based exercise programs for community-dwelling older adults in rural/regional areas: a scoping review
Source: Aging Clin Exp Res. 2022 Feb 12;34(7):1511–28. doi: 10.1007/s40520-022-02079-y (PMC8852913; doi:10.1007/s40520-022-02079-y)
Supplement: Supplementary file 1 — Supplementary file1 (PDF 118 KB) [file 40520_2022_2079_MOESM1_ESM.pdf]

Supplementary 1. The methods and findings for the comparison sample for each study.

Authors	Comparison Group						
	Comparison type	Total Sessions (frequency)	Session Duration (minutes)	Exercise Type	Specific Exercises	Delivery-Mode	Adherence
Hasegawa, Suzuki, Yamauchi	N/A	N/A	N/A	N/A	N/A	N/A	N/A
Jang et al.	Same exercise program - but with prefrail older adults	No sessions - coaching consisted of notification messages sent to wearable device with step count goals.	N/A	Walking	N/A	Externally, via wearable device	N/A
Jindo et al.	Same exercise program - but with no pedometer	11 (1/week)	90	Square-stepping	Warm-up (15 minutes), square stepping exercise (40 minutes), a recreational activity (20 minutes), and cool-down (15 minutes)	In-person group sessions	72.7% - 100% (97%)
Jindo et al.	N/A	N/A	N/A	N/A	N/A	N/A	N/A
Lin, Hwang, Wang, Chang & Wolf	Educational program only	N/A	N/A	N/A	N/A	N/A	N/A
McMahon et al.		N/A	N/A	N/A	N/A	N/A	N/A
Muscari et al.	Participants provided with educational materials about suggestions to improve lifestyle, including individualised self-administered programs to increase physical activity	N/A	N/A	N/A	N/A	N/A	N/A
Nicholson et al.	N/A	N/A	N/A	N/A	N/A	N/A	N/A
Okubo et al.	Balance training and muscle strengthening	No sessions-encouraged to do 3-5 days/week	60	Tai Chi and Muscle Strengthening	24-form Tai Chi, one leg stance, muscle strengthening targeting ankle dorsiflexors, plantar flexors, knee extensors, knee flexors, hip abductors, squats and lunges	Individual	73.30%
Okumiya et al.	N/A	N/A	N/A	N/A	N/A	N/A	N/A
Shigematsu et al.	Walking	12 (1/week)	40	Walking	Long distance outdoor walk	In-person group sessions	84.2% (SD = 23.7%)
Shigematsu et al.	Strength and Balance	24 (2/week)	70min	Strength and Balance	Warm-up (15 minutes), Exercises: squat, front lunge, leg extension, hip ab- duction and adduction, calf raise, abdominal curl, and back extension, one-leg and two-leg balance with the toes or heels raised, tandem	In-person group sessions	56%-92% (83%)

					standing/walking with eyes open/closed (standing position), and balancing the body on the buttocks with the feet off the ground (seated position) (40min) and cool-down (15 minutes)		
Sowie et al.	N/A	N/A	N/A	N/A	N/A	N/A	N/A
Tarazona-Santabalbina et al.	Received nutritional education	N/A	N/A	N/A	N/A	N/A	N/A
Wang et al	Seniors health promotion program; delivered by Community peer supporters	48 (2/week)	120	Seniors health promotion program delivered by senior nurses	Education; how to: choose a healthy diet, maintain oral hygiene, prevent falls, engage in physical activity, self-protect, be responsible for health, manage stress, and use resources (90min), mild exercise (30min)	In-person group	Not reported
Watanabe et al.	Unsupervised comprehensive geriatric intervention program	No sessions- encouraged to do 3-5 days/week	90	Resistance and strength training	low-load resistance exercises using body weight and/or elastic band, physical activity increments (up to 2500 steps/day from baseline), oral functional care, and a nutritional guide	At home	50%-100% (100%)
Yates and Dunnagan	N/A	N/A	N/A	N/A	N/A	N/A	N/A
Snapp, Malkin and Lloyd	Miscellaneous exercises	24 (3/week)	30	Miscellaneous exercises and stretching	Breathing and stretching exercises, sit-ups, leg raises, toe raisers, heel raisers, waist turning with ball, volleyball tip to each other, ball bounce to each other, kick the ball to each other and pickup ball and pass	In-person group	Not reported
