# Supplementary material for: Characteristics of community-based exercise programs for community-dwelling older adults in rural/regional areas: a scoping review
Source: Aging Clin Exp Res. 2022 Feb 12;34(7):1511–28. doi: 10.1007/s40520-022-02079-y (PMC8852913; doi:10.1007/s40520-022-02079-y)
Supplement: Supplementary file 2 — Supplementary file2 (PDF 99 KB) [file 40520_2022_2079_MOESM2_ESM.pdf]

Supplementary 2. Limitations stated for each study.

Authors	Limitations
Hasegawa, Suzuki, Yamauchi	Small sample size, not considering related factors to level of physical activity such as social/psychological and environment factors including public transport
Jang et al.	Small sample size and the study excluded frail older adults
Jindo et al.	The use of a specific exercise program as the intervention, relatively short testing period, not a randomised controlled trial, limited number of male participants and the study did not assess the actual physical activity for each group
Jindo et al.	The use of a specific exercise program as the intervention, not exploring the reasons why participants change their physical activity level through the study, only measured physical activity through pedometers not accounting for non-walking activities and a relatively younger population compared to the comparison
Lin, Hwang, Wang, Chang & Wolf	Participants being of poorer health were less likely to have completed telephone contact and some unmeasured behavioural characteristics and environmental factors also may have confounded and biased the study results
McMAhon et al.	Small sample size, study design limiting the analysis of results and not including long term follow up
Muscari et al.	MMSE as a result does not allow an adequate evaluation of exercise, due to the exclusion criteria a convenient subsample of the parent study was used, no dietary control and a relatively large amount of missing data due to the older population
Nicholson et al.	The groups were self-selected and the assessor was not blinded to the participants, small sample size, the cohort was indicated to be largely made up of non-fallers and no comparison training group was used
Okubo et al.	No blinding was applied, risk of overestimation because an intention to treat analysis was not available, a non-exercise group could not be studied and there was a low reliability of the trip data
Okumiya et al.	None reported
Shigematsu et al.	Short follow up period, inclusion of persons with a low fall risk, increase risk of false-positivity findings due to statistical analysis of the 15 outcome measures was performed separately and possible incorrect interpretation of and compliance with the prescribed exercise regimen
Shigematsu et al.	Low participation rate compared to those who received invitation to participate, small sample size and short follow up time
Snapp, Malkin and Lloyd	Small sample size, high attrition rate, not accounting for other potential result effecting variables and the use of a convenience sample
Sowie et al.	High quantity of white female participants, the study did not report on cognition or education levels, there was no control group to compare to and all data was self-reported
Tarazona-Santabalbina et al.	None reported
Wang et al	Selection bias was apparent due to the need of participants with certain health-related behaviours, there was no control group, potential threats to internal validity of the instrumentation and the potential of the Hawthorne effect
Watanabe et al	No control group, measurements were not blind to group allocation and cannot confirm if the same results can be translated to long term results
Yates and Dunnagan	Small sample size, limited number of participants utilising the nutrition offering, cannot establish whether the findings correlate to longer term results
